# Editorial: Developing biocompatible and biotechnological tools against cancer and autoimmune disorders

**DOI:** 10.3389/fimmu.2024.1466011

**Published:** 2024-08-01

**Authors:** Furkan Ayaz

**Affiliations:** ^1^ Department of Molecular Biology and Genetics, Faculty of Engineering and Natural Sciences, Biruni University, Zeytinburnu, Türkiye; ^2^ Molecular and Medicinal Biology Graduate Studies Programme, Institute of Health Sciences, Biruni University, Zeytinburnu, Türkiye

**Keywords:** immunotherapy, mRNA-based vaccines, autoimmune disorders, cancer immunotherapy, mRNA based immunotherapy, liposomes, drug delivery vehicles, medicinal biotechnology

In this Research Topic, we released a call for studies covering, biocompatible and biotechnological molecules that can be utilized in the therapeutic approaches of versatile diseases. Natural or synthetic chemical compounds are foreign to our bodies and this may cause unwanted toxicity during the treatment process. With the advancement in the field now, we can use biomolecules such as peptides, antibodies, nucleic acids and other biotechnological products as biotherapeutics at the clinical setting. Especially with the developments in the immunotherapy area, there are alternative and more effective tools against cancer, autoimmune, allergic and inflammatory disorders. The main and overarching aim of this Research Topic was modulation of the immune system components in the line of our needs by using biotechnological tools or biomolecules that are biocompatible to our bodies.

As part of this Research Topic, we were expecting the leading researchers to share their findings in this area, to further pave the way for the productive minds in the area. Original research and Review articles that focus on the novel biomolecule based immunotherapy applications were received for the reviewer evaluation in this Research Topic. Data on the utilization of nucleic acids, peptides, vectors, antibodies, and proteins as well as their delivery vehicles either in a liposome or another biocompatible form were primarily in the scope of this article collection. We aim to present the latest research in the field of immunotherapy to further expand our vision and generate greater interest in this field to develop innovative treatment strategies for cancer and autoimmune disorders.

In the original article of Vuscan et al., the team is presenting data regarding the utilization of *Saccharomyces cerevisiae*’s β-glucans’ effect on the monocyte function and eventually anti-tumor activity. In their comparative analysis of the β-glucans from different fractions and strains, they found the most potent immunostimulator of the monocytes. They measured the production of different pro-inflammatory cytokines and compared them to the control groups. Moreover, their analysis also focused on the deciphering the intracellular signaling pathways that were activated by these biomolecules. They further tested this effect on the *in vivo* murine models of the cancer. Bladder cancer and melanoma tumor growth were reduced with the administration of the β-glucan that exerted the highest activity *in vitro*.

In the mini review study by Koch et al., the authors focused on the polymeric materials that can be utilized in autoimmune disorders. Bone marrow failure is the specific disease that they focused on in their study. In this autoimmune disorder the immune system cells mediate the elimination of the hematopoietic stem cells which leads to bone marrow failure eventually. Biocompatible polymeric compounds can be generated for the delivery of the immunosuppressive drug molecules to the patients. Moreover, novel biomaterial based polymeric compounds are generated to form a cell matrice nest for the limited stem cells that are isolated from the donors in order to extend the stem cells for transfer into the patients that are in need. All of these exciting topics are covered in more details in their mini review.

SERPINB9 (Sb9), Proteinase Inhibitor 9 (PI-9), is a proteinase inhibitor that specifically inhibits Granzyme B (GzmB). GzmB is the inducer of the apoptosis and is secreted by cytotoxic T and natural killer cells. Due to regulatory function of Sb9 on the GzmB, it is a vital target in the modulation of the immune response in versatile disease settings from infection to autoimmunity to cancer. Huang et al. filtered through the recent studies and findings in their review study to further highlight the therapeutic potential of Sb9 based formulations. In their study, they firstly focus on the biology of Sb9 and its distribution among different cell types. Then they further delve into the details of the nanoparticle based approaches targeting Sb9. They present the top-notch studies targeting Sb9 with versatile molecular tools for immunotherapy purposes.

In their original article Kim et al. present a novel humanized mice model that can be utilized in the cancer immunotherapy, virology and transplantation studies. Mouse models are easy to study but with human cell samples the scientists face difficulties due to the rejection by the mouse immune system. Moreover, the human cells mimic the human diseases much better than the mouse cell based models. Especially in cancer models the field has been facing issues. In their study, the authors generated Rag2 null IL2rγ null mice that was preconditioned either with irradiation or busulfan treatment before repopulation of their bone marrow with human hematopoietic stem cells. This valuable tool will have major impact in the field while studying mouse models of the human diseases.

We believe that this Research Topic will grasp the attention of the readers in the field. The studies in this Research Topic will have major impact in the field to further our knowledge in biomolecule and biotechnological tool based immunotherapy approaches in cancer, autoimmune, inflammatory and allergic disorders.

## Author contributions

FA: Conceptualization, Validation, Writing – original draft, Writing – review & editing.

